# Endothelial glycocalyx is damaged in diabetic cardiomyopathy: angiopoietin 1 restores glycocalyx and improves diastolic function in mice

**DOI:** 10.1007/s00125-022-05650-4

**Published:** 2022-02-25

**Authors:** Yan Qiu, Stanley Buffonge, Raina Ramnath, Sophie Jenner, Sarah Fawaz, Kenton P. Arkill, Chris Neal, Paul Verkade, Stephen J. White, Melanie Hezzell, Andrew H. J. Salmon, M.-Saadeh Suleiman, Gavin I. Welsh, Rebecca R. Foster, Paolo Madeddu, Simon C. Satchell

**Affiliations:** 1 Bristol Renal, Bristol Heart Institute, Translational Health Sciences, University of Bristol, Bristol, UK; 2 Biodiscovery Institute, Medicine, University of Nottingham, Nottingham, UK; 3 School of Biochemistry, University of Bristol, Bristol, UK; 4 Department of Life Sciences, Manchester Metropolitan University, Manchester, UK; 5 Bristol Veterinary School, University of Bristol, Langford, UK; 6 Renal Service, Specialist Medicine and Health of Older People, North Shore Hospital, Waitemata District Health Board, Takapuna, Auckland, New Zealand; 7 Bristol Heart Institute, Translational Health Sciences, University of Bristol, Bristol, UK

**Keywords:** Angiopoietin 1, Coronary microcirculation, Diabetes, Glycocalyx, Permeability

## Abstract

**Aims/hypothesis:**

Diabetic cardiomyopathy (DCM) is a serious and under-recognised complication of diabetes. The first sign is diastolic dysfunction, which progresses to heart failure. The pathophysiology of DCM is incompletely understood but microcirculatory changes are important. Endothelial glycocalyx (eGlx) plays multiple vital roles in the microcirculation, including in the regulation of vascular permeability, and is compromised in diabetes but has not previously been studied in the coronary microcirculation in diabetes. We hypothesised that eGlx damage in the coronary microcirculation contributes to increased microvascular permeability and hence to cardiac dysfunction.

**Methods:**

We investigated eGlx damage and cardiomyopathy in mouse models of type 1 (streptozotocin-induced) and type 2 (*db*/*db*) diabetes. Cardiac dysfunction was determined by echocardiography. We obtained eGlx depth and coverage by transmission electron microscopy (TEM) on mouse hearts perfusion-fixed with glutaraldehyde and Alcian Blue. Perivascular oedema was assessed from TEM images by measuring the perivascular space area. Lectin-based fluorescence was developed to study eGlx in paraformaldehyde-fixed mouse and human tissues. The eGlx of human conditionally immortalised coronary microvascular endothelial cells (CMVECs) in culture was removed with eGlx-degrading enzymes before measurement of protein passage across the cell monolayer. The mechanism of eGlx damage in the diabetic heart was investigated by quantitative reverse transcription-PCR array and matrix metalloproteinase (MMP) activity assay. To directly demonstrate that eGlx damage disturbs cardiac function, isolated rat hearts were treated with enzymes in a Langendorff preparation. Angiopoietin 1 (Ang1) is known to restore eGlx and so was used to investigate whether eGlx restoration reverses diastolic dysfunction in mice with type 1 diabetes.

**Results:**

In a mouse model of type 1 diabetes, diastolic dysfunction (confirmed by echocardiography) was associated with loss of eGlx from CMVECs and the development of perivascular oedema, suggesting increased microvascular permeability. We confirmed in vitro that eGlx removal increases CMVEC monolayer permeability. We identified increased MMP activity as a potential mechanism of eGlx damage and we observed loss of syndecan 4 consistent with MMP activity. In a mouse model of type 2 diabetes we found a similar loss of eGlx preceding the development of diastolic dysfunction. We used isolated rat hearts to demonstrate that eGlx damage (induced by enzymes) is sufficient to disturb cardiac function. Ang1 restored eGlx and this was associated with reduced perivascular oedema and amelioration of the diastolic dysfunction seen in mice with type 1 diabetes.

**Conclusions/interpretation:**

The association of CMVEC glycocalyx damage with diastolic dysfunction in two diabetes models suggests that it may play a pathophysiological role and the enzyme studies confirm that eGlx damage is sufficient to impair cardiac function. Ang1 rapidly restores the CMVEC glycocalyx and improves diastolic function. Our work identifies CMVEC glycocalyx damage as a potential contributor to the development of DCM and therefore as a therapeutic target.

**Graphical abstract:**

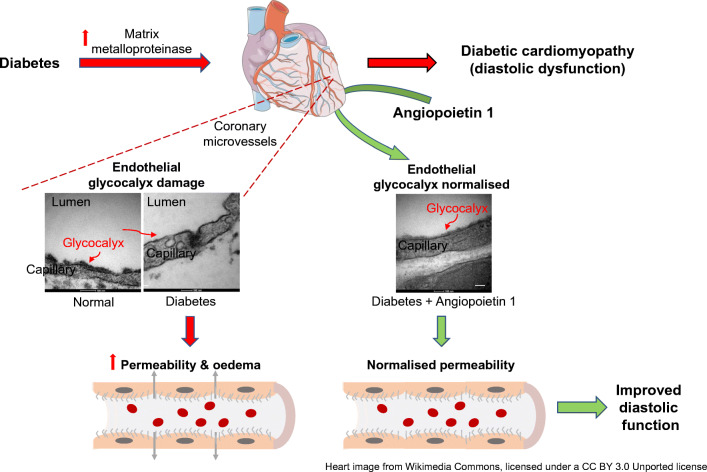

**Supplementary Information:**

The online version contains peer-reviewed but unedited supplementary material available at 10.1007/s00125-022-05650-4.

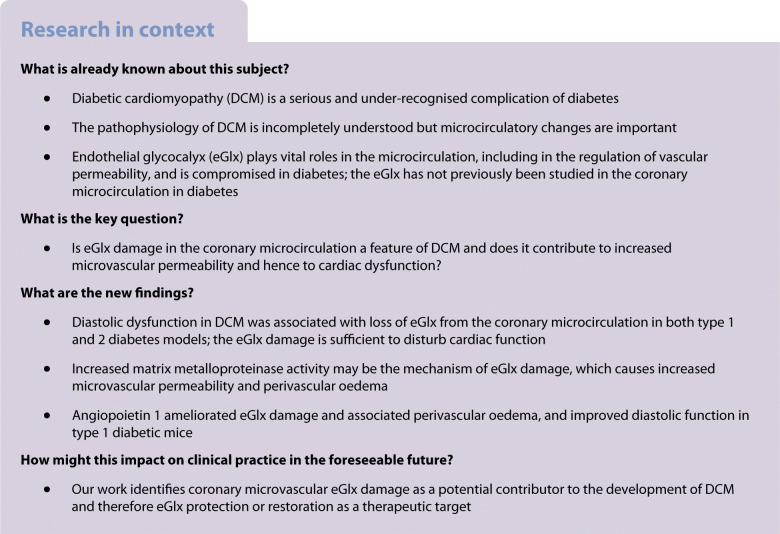



## Introduction

Diabetic cardiomyopathy (DCM) is a common and important complication of diabetes, characterised by progression from diastolic dysfunction to heart failure in the absence of coronary artery disease and hypertension. It is under-recognised, with up to 75% of young asymptomatic diabetic individuals having echocardiographic evidence of diastolic dysfunction [1]. DCM may evolve rapidly to global cardiac dysfunction with poor clinical outcomes [2]. Better understanding of the disease pathophysiology is urgently required to direct development of effective treatments. Deranged calcium homoeostasis, increased mitochondrial fatty acid oxidation, myocardial fibrosis, upregulation of proinflammatory cytokines, increased activation of the systemic and tissue renin–angiotensin–aldosterone system, as well as deranged ubiquitin proteasome system have been suggested to be involved in the development of DCM [3]. The association of microvascular dysfunction with DCM suggests that it may be involved in disease pathogenesis [4–6]. Indeed, the importance of generalised microvascular disease in diabetes is well recognised [7] but the role of the microcirculation in cardiac pathophysiology is understudied [8]. In the coronary microcirculation, hyperglycaemia causes endothelial dysfunction, increased permeability and oedema [9].

The heart is extremely sensitive to minor changes in vascular permeability. Ensuing oedema not only expands the interstitial compartment thereby increasing the diffusion distance for oxygen and other nutrients, which may compromise cellular metabolism, but also directly affects diastolic relaxation by increasing ventricular stiffness. Only a few per cent increase in interstitial fluid volume compromises cardiac function [10].

The endothelial glycocalyx (eGlx) is a hydrated polyanionic gel, found on the luminal surface of all endothelial cells, composed of proteoglycans and sialoproteins [11, 12]. Proteoglycans consist of a core protein (e.g. syndecan 4 [SDC4]) and glycosaminoglycan (GAG) side chains. Heparan sulphate and chondroitin sulphate GAGs are prominent in the eGlx. Hyaluronan is a non-sulphated GAG that may be anchored to the cell surface by specific receptors and interacting proteins or simply adsorbed onto the cell surface. The eGlx has multiple roles in vascular physiology and its disruption contributes to several diseases [13, 14]. Importantly for this project, eGlx plays key roles in the homoeostatic maintenance of vascular permeability in different vascular beds, including the coronary microvasculature [11, 12, 15, 16], and microvascular perfusion [17].

We have shown that diabetic conditions, including high glucose concentrations [18], reactive oxygen species [19] and inflammatory mediators [20], damage the eGlx in vitro, resulting in increased permeability [18, 19]. The eGlx is lost from the glomerular circulation in animal models of diabetes and this can be ameliorated by soluble mediators and matrix metalloproteinase (MMP) inhibitors, with functional improvement in albuminuria [16] and glomerular permeability [21]. We have found that the Tie2 receptor agonist angiopoietin 1 (Ang1) restores eGlx in enzyme-treated mesenteric microvessels [22] and in glomeruli from diabetic rats [21]. In human diabetes there is a correlation between the loss of systemic eGlx and increased microvascular permeability evidenced by proteinuria [23].

We therefore hypothesised that coronary microvascular endothelial cell (CMVEC) glycocalyx damage would occur in diabetes, leading to increased coronary microcirculation permeability and development of ventricular dysfunction, and furthermore that restoration of the eGlx would protect against microvascular permeability and ventricular dysfunction.

## Methods

For detailed methods, please refer to the electronic supplementary material (ESM) Methods.

### Cell culture and treatment

Primary human CMVECs (Lonza, Basel, Switzerland) were cultured in endothelial growth medium 2 microvascular (EGM2-MV; Lonza) containing 5% vol./vol. FCS and growth factors as supplied, with the exception of vascular endothelial growth factor (VEGF), at 37°C. We used human conditionally immortalised CMVECs (ciCMVECs; ESM Fig. 1), generated using a similar approach as used previously in glomerular endothelial cells [24]. Briefly, primary human CMVECs were transduced with temperature-sensitive simian virus 40 large tumour (tsSV40LT) antigen and telomerase using retroviral vectors. At the permissive temperature of 33°C, the tsSV40LT transgene is activated, causing cell proliferation (without telomere shortening), whereas at 37°C, the transgene is inactivated, rendering cells non-proliferative and quiescent. The ciCMVECs were cultured in the same medium as for primary CMVECs and used for experiments after 5 days at the non-permissive temperature.

### Protein extraction and western blotting

To characterise ciCMVECs, monolayers of primary CMVECs, undifferentiated ciCMVECs and differentiated ciCMVECs were lysed in lysis buffer. Protein in the supernatant fraction was used for western blotting. Protein samples were probed with antibodies against the endothelial markers CD31, vascular endothelial cadherin (VE-cadherin) and VEGF receptor 2 (VEGFR2).

### Animal models of diabetes

Experiments were performed in accordance with the Guide for the care and use of laboratory animals, eighth edition (2011). All animal procedures performed conformed to the guidelines from Directive 2010/63/EU of the European Parliament on the protection of animals used for scientific purposes and were approved by the University of Bristol and the British Home Office (licenses: PPL 30/2811 and PPL 30/3373).

Type 1 diabetes was induced in male FVB mice (TG 287 SATOJ; The Jackson Laboratory, USA) by injection of streptozotocin (STZ; 50 mg/kg body weight per day, i.p. for five consecutive days) [6]. Control mice received citric acid buffer. All mice were fasted for 4–6 h before administration of STZ. Hyperglycaemia was confirmed by blood glucose levels of >16 mmol/l on two consecutive days. Only mice remaining hyperglycaemic over the duration of the study were included. Body weight and blood glucose were monitored weekly with a glucose meter (ACCU-CHEK Aviva; Roche, UK) from 1 week after administration of STZ until the end of the study (9 weeks after STZ injection).

In addition, male leptin-receptor mutant *db*/*db* mice (BKS.Cg − +*Lepr*^db^/*Lepr*^db^/OlaHsd; Harlan, UK) were used as a model of insulin-resistant type 2 diabetes. Increases of blood glucose begin at 6 weeks of age and diastolic dysfunction from 9 weeks in these mutant mice [6]. Age-matched lean mice (BKS.Cg-*m*^+/+^*Lepr*^db^/OlaHsd) were used as control. Two groups of mutant mice were studied in this project, one at early diabetes (7 weeks old) and the other at a later (12 weeks old) time point. Hyperglycaemia was assessed as above.

### Haemodynamic measurements

Diastolic dysfunction is the major phenotype in the development of DCM [25]. Diastolic function was determined by both E/A ratio with pulsed wave Doppler [6, 26] and E/E′ with tissue Doppler [27] using a high-frequency, high-resolution echocardiography system (Vevo 3100; VisualSonics, Toronto, Canada). Images were captured at a heart rate of 380 ± 10 beats/min under 1–3% vol./vol. isoflurane anaesthesia. Systolic function was determined with the analysis of M-mode images at a heart rate of 450 ± 30 beats/min. Mice were excluded from the experiments if their heart rates did not reach the optimal range for the measurement of either diastolic or systolic function within 30 min after initiating monitoring.

### Determination of eGlx depth and coverage, perivascular space and endothelial cell thickness

To quantify eGlx parameters by transmission electron microscopy (TEM), mouse hearts were stopped at the end of diastole by the injection of 2.5 ml of 0.1 mol/l CdCl_2_ and perfusion-fixed for electron microscopy preparation as previously described [16]. To study CMVEC glycocalyx, four capillaries were analysed at random from each left ventricle section (one per mouse). TEM images were acquired and eGlx depth and coverage were determined using ImageJ as described previously [16]. Perivascular space area was determined using ImageJ and presented as a proportion of the area of the capillary lumen [28].

### Trichrome staining to identify perivascular fibrosis

To investigate whether fibrosis formation accounts for the increased perivascular space in diabetic FVB mice, mouse heart sections were stained with Trichrome Stain (Masson) Kit (Sigma Aldrich, UK) according to the manufacturers’ instructions.

### Localising lectin-binding molecules using correlative light and electron microscopy in mouse and human tissues

Until now, eGlx labelling has relied on perfusion fixation techniques which are technically demanding, require the use of a whole animal and are limited to animal samples. We developed a novel technique to localise lectin binding to the eGlx in immersion-fixed samples using correlative light and electron microscopy. Mice were perfusion-fixed with 4% wt/vol. paraformaldehyde (PFA) in PBS, and hearts were dissected and embedded in paraffin. Sections of normal human myocardium were obtained from the Bristol Coronary Biobank (ethical approval 08/H107/48). Sections were incubated with a range of biotinylated lectins, binding to different carbohydrate residues. After washing, quantum dots 655 (6× 12 nm)-conjugated streptavidin (ThermoFisher Scientific, UK) was applied for 1 h at room temperature. Fluorescence images were captured to confirm specific staining. Sections were then prepared for imaging with an FEI Tecnai 12 (120KV BioTwin Spirit) transmission electron microscope (FEI, Cambridge, UK).

### Lectin-based fluorescence and immunofluorescence to quantify eGlx and its component

Tissue sections were prepared as above. After sections were incubated with biotinylated lectins, 2 μg/ml of Alexa Fluor 488-conjugated streptavidin was applied for 1 h at room temperature. DAPI was applied for counterstaining.

For SDC4 expression, 5 μm thick frozen heart sections were fixed with 4% PFA. The sections were incubated with primary antibodies (purified rat anti-mouse SDC4, Clone KY/8.2 from BD Biosciences, UK and VE-cadherin, SC-9989, mouse anti-mouse, from Santa Cruz Biotechnology, Germany, at a 1:50 dilution) in 1% BSA wt/vol. overnight at 4°C after blocking. After washing, sections were incubated with Alexa Fluor 488 anti-rat and Alexa Fluor 546 anti-mouse secondary antibodies (Thermo Fisher Scientific, UK), followed with counterstaining with DAPI. Images were captured using a Leica SP5-II confocal laser scanning microscope (Leica Microsystems, UK) and analysed with Coloc 2 in Fiji. Colocalisation was presented as the proportion of the VE-cadherin-stained area that also had SDC4 staining.

### Measurement of transendothelial protein passage

Twelve-millimetre transwell (0.4 μm pore polyester membrane insert, 1.12 cm^2^ surface area; Corning, UK) were seeded with ciCMVECs at 37,500 cells/cm^2^. Cells were cultured at 33°C for 2 days and 37°C for 5 days. Media were changed three times a week. Transendothelial permeability to macromolecules was assessed by measurement of passage of Alexa Fluor 488-conjugated BSA (ThermoFisher, UK) across the monolayer in tissue culture inserts [29]. The cells were starved of serum for 2 h and then treated with a combination of enzymes (heparinase 1 U/ml + hyaluronidase 4.5 U/ml + chondroitinase 100 mU/ml) for 3 h to remove eGlx, before BSA passage measurement.

### Fluorescence labelling

To confirm eGlx removal, ciCMVECs were fixed and stained with FITC-labelled wheatgerm agglutinin (WGA), as described previously [19]. To confirm that the cell monolayer remained intact after enzyme treatment, cells were stained with anti-VE-cadherin as described previously [18].

### Fluorescence activated cell sorting

To identify the genes relevant to eGlx synthesis and shedding, heart endothelial cells were collected by fluorescence activated cell sorting (FACS), as described previously [20], from control and diabetic FVB mice 9 weeks after STZ injection. Briefly, left ventricles were enzymatically digested to give a single-cell suspension, immunostained with phycoerythrin rat anti-mouse CD31 antibody and propidium iodide to exclude dead cells. FACS was carried out on the live cells.

### TaqMan qRT-PCR array

Total RNA was extracted from heart endothelial cells collected by FACS from control and diabetic FVB mice 9 weeks after STZ injection. We designed a quantitative reverse transcription-PCR (qRT-PCR) array including 96 glycocalyx-related and endothelial genes [20]. For the selected genes of interest, the ${2}^{-\Delta \Delta {\mathrm{C}}_{\mathrm{t}}}$ method was also used to calculate fold changes, normalised to the geometric mean of 18 s and β-actin.

### MMP activity assay

Left ventricle was dissected from control and diabetic FVB mice 9 weeks after STZ injection. Total protein was extracted. MMP2 Biotrak Activity Assay (GE Healthcare Life Sciences, UK) and MMP9 ELISA (AnaSpec, Fremont, CA, USA) were carried out according to the manufacturers’ instructions. The concentrations of active MMP2 and MMP9 were normalised to total protein.

### Langendorff preparation to measure effects of enzymatic eGlx removal

Seven-week-old male Sprague Dawley rats (approximately 250 g; Harlan, Bicester, UK) were euthanised by cervical dislocation and hearts quickly removed, mounted on a Langendorff apparatus and perfused in a non-recirculating mode with Krebs solution as described previously [30]. Contractile function was measured using a latex balloon in the left ventricle. Data acquisition and analysis used a PowerLab System (AD Instruments, Bella Vista, NSW, Australia). Measurement was initiated after hearts were stabilised by perfusion for 30 min. Hearts were then perfused for 40 min with the combination of hyaluronidase (14 μg/ml; Sigma Aldrich, UK) and chondroitinase (0.0022 u/ml; Sigma Aldrich) in Krebs solution to deplete eGlx or with Krebs solution alone as control. We have shown previously that this combination of enzymes reduces eGlx thickness and coverage in glomerular capillaries [21]. Some isolated hearts were also perfusion-fixed with glutaraldehyde and Alcian Blue for analysing eGlx by electron microscopy as above.

### Restoration of eGlx in vivo

Rescue experiments were performed in the FVB mouse model of type 1 diabetes, aiming to restore the eGlx and reverse diastolic dysfunction, by a single injection of Ang1. Nine weeks after STZ injection, FVB mice were randomised to receive 100 μl of Ang1 (i.v. through the retro-orbital vein to achieve 200 ng/ml of blood volume) or vehicle (PBS, 100 μl) and diastolic function was assessed by echocardiography. Other mice received the same treatments and were perfusion-fixed for electron microscopy and eGlx analysis at 1 h or 3 h after Ang1 injection.

### Statistical analysis

All statistical analyses were conducted with Prism version 5.00 (GraphPad Software, USA) (*p< 0.05, **p< 0.01, ***p< 0.001). All data are expressed as mean ± SEM and all *n* numbers represent biological repeats. A Student’s two-tailed *t* test was used to determine the significance of the difference between means of two groups and Pearson *r* test was used to determine how strongly two groups of data related when data passed a normality test. A normal distribution of the data was tested using the Kolmogorov–Smirnov test if the sample size allowed. If normal distribution or equal-variance assumptions were not valid, statistical significance was evaluated using the Mann–Whitney test for two groups or Spearman *r* test for correlation. Mice were randomly assigned to treatment groups. Tested samples were assayed in a blinded fashion.

## Results

### Diastolic dysfunction is associated with loss of eGlx in a mouse model of type 1 diabetes

**Fig. 1:**
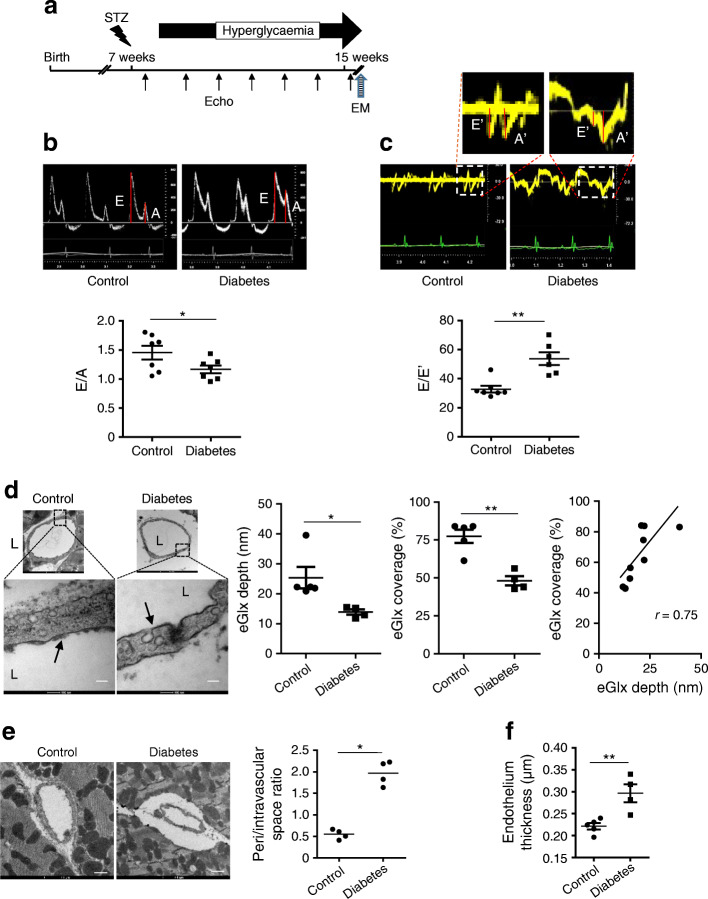
EGlx damage is associated with the development of DCM in FVB mice. (**a**) Diabetes was induced in FVB male mice by injection of low doses of STZ. The development of DCM was monitored with echocardiography for 9 weeks after STZ injection. (**b**, **c**) Representative pulsed wave Doppler images and reduced E/A ratio (**b**) and representative tissue Doppler images and increased E/E′ (**c**) in mice 9 weeks after STZ injection compared with control mice (*n* = 7 for control and 6 for diabetes; *p< 0.05 and **p< 0.01 [unpaired *t* test]). (**d**) Reduced eGlx depth and coverage in mice with DCM (*n* = 5; *p< 0.05 [unpaired *t* test]). Low- and high-magnification electron micrographs of capillaries from left ventricles of STZ-injected and control mice 9 weeks after STZ injection. Arrows show eGlx on top of endothelial cells. Scale bar, 100 nm. eGlx coverage (percentage of grid points at which eGlx depth is not less than 10 nm) is strongly positively associated with eGlx depth (*n* = 9; *p< 0.05 [Pearson *r* = 0.75]). (**e**, **f**) Increased peri/intravascular space ratio (area of perivascular space/area of intravascular space; *n* = 4; *p< 0.05 [unpaired *t* test]) (**e**) and endothelial thickness (*n* = 4 or 5; **p< 0.01 [unpaired *t* test]) (**f**) in the capillaries from diabetic vs non-diabetic hearts. Scale bar, 1 μm. Data are presented as mean ± SEM. EM, electron microscopy; L, capillary lumen

STZ-treated FVB mice became diabetic as expected (Table 1). A significant decrease in E/A ratio and increase in E/E′ (Fig. 1b,c, Table 2), indicating diastolic dysfunction, was observed at 9 weeks after STZ injection. No systolic dysfunction was detected in diabetic mice (ESM Fig. 2). Hearts were processed for electron microscopy after perfusion fixation with glutaraldehyde and Alcian Blue. eGlx depth in left ventricular capillaries, 2–5 μm in diameter, was significantly decreased (by 45%) in diabetic mice (Fig. 1d). eGlx coverage was also reduced (by 39%) in diabetic mice and was significantly correlated with eGlx depth.

**Table 1:** Mouse tail-vein blood glucose levels after STZ injection

Mouse	Time after STZ injection		
	2 weeks	6 weeks	9 weeks
Control (mmol/l, *n* = 7)	9.92 ± 0.44	9.75 ± 0.43	9.15 ± 0.38
STZ (mmol/l, *n* = 6)	29.27 ± 1.42***	32.04 ± 0.82***	33.00 ± 0***

Data are presented as mean ± SEM

***p< 0.001 vs control

**Table 2:** Pulsed wave and tissue Doppler parameters in FVB mice 9 weeks after STZ injection

Mouse	Pulsed wave Doppler			Tissue Doppler		
	E (mm/s)	A (mm/s)	IVRT (ms)	E’ (mm/s)	A’ (mm/s)	E’/A’
Control (*n* = 7)	697 ± 39	489 ± 28	27.8 ± 1.8	21.5 ± 1.1	18.0 ± 1.5	1.26 ± 0.13
Diabetes (*n* = 6)	636 ± 55	554 ± 32	33.5 ± 1.6*	12.4 ± 1.6***	22.4 ± 2.3	0.555 ± 0.062***

Data are presented as mean ± SEM

*p< 0.05 and ***p< 0.001 vs control

IVRT, isovolumic relaxation time

Having shown eGlx damage in left ventricular capillaries of diabetic mice, we measured the perivascular space as an index of oedema [28, 31]. Figure 1e shows that the perivascular space in diabetic FVB mice was increased 2.7-fold. The endothelium was significantly thickened (by 32%) in the left ventricular capillaries (Fig. 1f), confirming endothelial damage [32–34]. There was no evidence of an increase in perivascular fibrosis (ESM Fig. 3).

### Lectin-based imaging confirms loss of eGlx in a mouse model of type 1 diabetes and demonstrates eGlx in human coronary capillaries

**Fig. 2:**
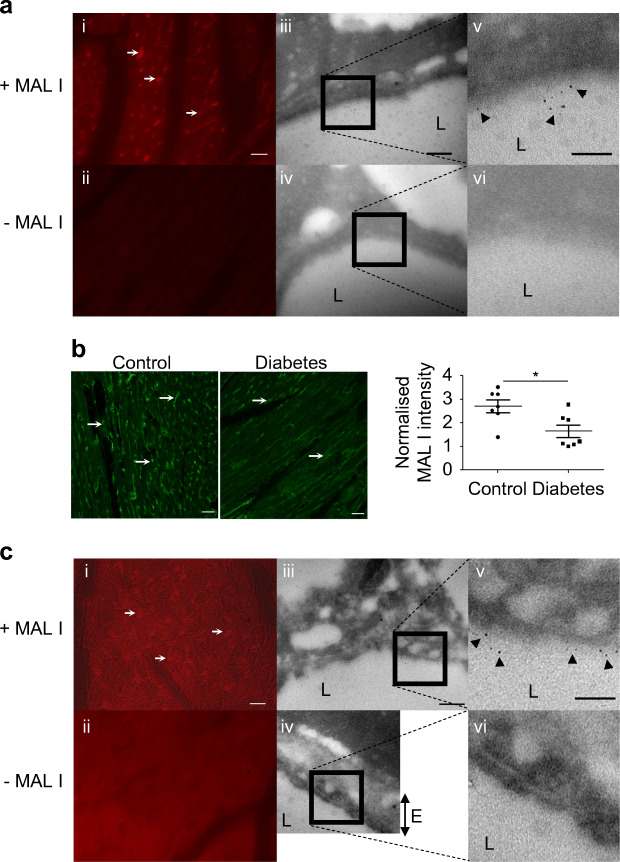
MAL I lectin staining confirms loss of eGlx in a mouse model of type 1 diabetes and demonstrates eGlx in human coronary capillaries. (**a**) MAL I lectin binds to sugar residues in the eGlx of FVB mouse coronary capillaries (i, ii show fluorescence microscopy images; iii–vi show TEM images). MAL I lectin-binding molecules mainly expressed in eGlx were confirmed by correlative light and electron microscopy using quantum dots binding to MAL I. Arrows point to coronary microvessels stained with MAL I. Arrowheads point to quantum dots associated with MAL I. (**b**) Reduced MAL I expression in left ventricles of mice with DCM (*n* = 7; *p< 0.05 [unpaired *t* test]). Arrows point to coronary microvessels stained with MAL I. (**c**) MAL I lectin binds to sugar residues in eGlx of human coronary capillaries (i, ii show fluorescence microscopy images; iii–vi show TEM images). Arrows point to coronary microvessels stained with MAL I. Arrowheads point to quantum dots associated with MAL I. (**a**, **c**) Scale bar, 15 μm in fluorescent images, 200 nm in electron microscopy images **a**(iii, iv) and **c**(iii, iv), and 100 nm in electron microscopy images **a**(v, vi) and **c**(v, vi). (**b**) Scale bars, 25 μm. Data are presented as mean ± SEM. E, endothelium; L, capillary lumen

The suitability of a panel of lectins to label eGlx was investigated. Figure 2a(i, ii) shows fluorescence microscopy images of quantum dots labelled with *Maackia amurensis* lectin I (MAL I) lectin in a control mouse heart section. The same section was then processed for TEM to confirm localisation of the lectin in the eGlx. Paraffin sections enable both light and electron microscopy but are not optimal for the latter. However, the lumen and capillary walls were clearly visible, as shown in Fig. 2a(iii–vi). MAL I-associated quantum dots were present on the luminal surface of endothelial cells but not on the abluminal side or basement membrane, confirming that MAL I labels the eGlx [Fig. 2a(iii–vi)]. We found, in addition, that the lectins *Marasmium oreades* agglutinin (MOA), *Sambucus nigra* agglutinin (SNA) and *Lycopersicon esculentum* agglutinin (LEA) bound mainly to eGlx but that isolectin B4 (IB4) and WGA bound to both eGlx and basement membrane (ESM Fig. 4).

MAL I lectin was then used to quantify eGlx changes in left ventricular capillaries by fluorescence intensity. eGlx was significantly reduced in the left ventricular capillaries from mice with diabetes (Fig. 2b). Capillary density, measured by counting WGA-labelled vessels (ESM Fig. 5), was unchanged. We have previously observed decreased capillary density in type 1 diabetes in CD1 mice [6] but this observation was made at a much later time point (20 weeks) than examined here (9 weeks).

In human coronary capillaries MAL I bound to the eGlx (Fig. 2c), enabling visualisation of human CMVEC glycocalyx in tissue sections for the first time. MOA did not bind to human coronary vessels. IB4, WGA and LEA bound to both eGlx and basement membrane (ESM Fig. 6a) whereas SNA bound mainly to eGlx (ESM Fig. 6b) in human CMVECs.

### Enzymatic eGlx dysfunction causes increased transendothelial permeability and myocardial MMP activity is increased in diabetes

**Fig. 3:**
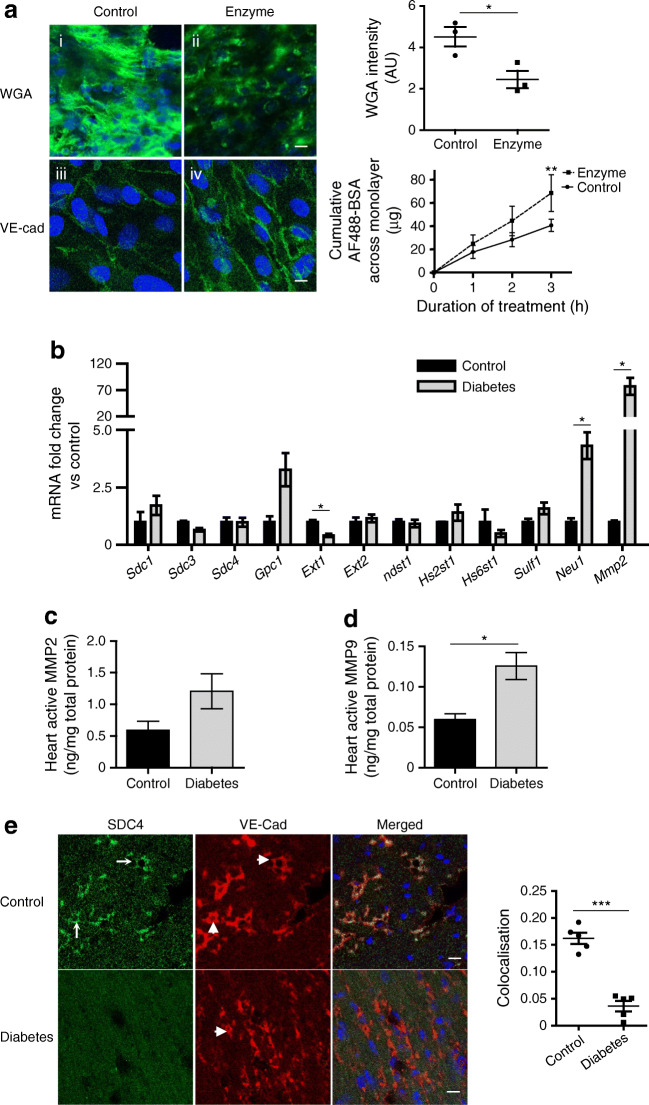
Enzymatic eGlx disruption causes increased transendothelial permeability and myocardial MMP activity is increased in diabetes. The direct effect of eGlx damage on endothelial cell function was investigated on ciCMVECs. ciCMVECs were cultured until cells formed a confluent monolayer, then subjected to enzyme treatment (heparinase 1 U/ml + hyaluronidase 4.5 U/ml + chondroitinase 100 mU/ml) in serum-free medium for 3 h before BSA passage measurement. (**a**) Enzymatic treatment reduced eGlx, confirmed by reduced FITC–WGA binding (*n* = 3; *p< 0.05 [paired *t* test]), and enhanced BSA passage across the ciCMVEC monolayer (*n* = 3; **p< 0.01 [two-way ANOVA]) (i, ii). The ciCMVEC monolayer cell–cell junctions remained intact, as shown by maintained VE-cadherin junctional staining (iii, iv). Scale bar, 10 μm. (**b**) The mRNA expression levels of enzymes relevant to eGlx synthesis and shedding were investigated with TaqMan qRT-PCR array on endothelial cells collected by FACS from FVB mouse hearts 9 weeks after STZ injection. *Mmp2* mRNA level was dramatically increased (*n* = 3; *p< 0.05 [unpaired *t* test]). (**c**, **d**) MMP2 (**c**) and MMP9 (**d**) activity in FVB mouse heart tissue homogenate were measured by activity assays. We found a significant increase in MMP9 activity in diabetic heart (*n* = 4; *p< 0.05 [unpaired *t* test]) and a non-significant increase in MMP2 activity (*p* = 0.11). (**e**) SDC4 was mainly expressed in coronary microvessels and its expression was reduced in left ventricles of mice with DCM (*n* = 5; ***p< 0.001 [unpaired *t* test]). Arrows point to SDC4 staining and arrowheads point to VE-cadherin staining. Scale bar, 10 μm. Data are presented as mean ± SEM. AF488, Alexa Fluor 488; AU, arbitrary units; VE-Cad, VE-cadherin

The direct effect of eGlx loss on endothelial barrier function was investigated using ciCMVEC monolayers. Enzymatic treatment reduced eGlx, confirmed by reduced FITC–WGA binding, and enhanced BSA passage across the CMVEC monolayer (Fig. 3a), indicating increased cell permeability to macromolecules. The CMVEC monolayer cell–cell junctions remained intact, as shown by maintained VE-cadherin junctional staining.

To investigate the mechanism underlying eGlx damage, we performed a qRT-PCR array focused on glycocalyx-related genes (Table 3) on CMVECs collected by FACS from type 1 diabetic mice. The mRNA for MMP2, an enzyme involved in eGlx shedding, was increased by over 70-fold in diabetic mice (Fig. 3b). We further investigated MMP2 and MMP9 activity in left ventricle homogenate since MMP2 and MMP9 have similar actions in eGlx shedding. We found a significant increase in MMP9 activity and a non-significant increase in MMP2 activity (*p* = 0.11) in diabetic hearts (Fig. 3c,d).

**Table 3:** List of genes in TaqMan array relevant to heparan sulphate synthesis and shedding

Symbol	Name	Class
*Sdc1*, *Sdc3*, *Sdc4*	Syndecan 1, 3, 4	Proteoglycan
*Gpc1*	Glypican 1	Proteoglycan
*Ext1*, *Ext2*	Exostoses 1, 2	Heparan sulphate-synthesising enzyme
*Ndst1*	*N*-deacetylase/*N*-sulfotransferase 1	Heparan sulphate-synthesising enzyme
*Hs2st1*	Heparan sulphate 2-*O*-sulfotransferase 1	Heparan sulphate-synthesising enzyme
*Hs6st1*	Heparan sulphate 6-*O*-sulfotransferase 1	Heparan sulphate-synthesising enzyme
*Sulf1*	Sulphatase 1	Heparan sulphate-degrading/modifying
*Neu1*	Neuraminidase 1/sialidase 1	Sialic acid-modifying enzyme
*Mmp2*, *Mmp9*	Matrix metalloproteinase 2, 9	Glycocalyx sheddases

SDC4 is a major component of the eGlx and its expression in the myocardium was largely restricted to coronary microvessels, confirmed by colocalisation with the endothelial marker VE-cadherin. SDC4 protein levels were dramatically reduced in diabetic CMVECs (Fig. 3e), consistent with eGlx shedding.

### Diastolic dysfunction is associated with and preceded by eGlx reduction in a *db*/*db* mouse model of type 2 diabetes

**Fig. 4:**
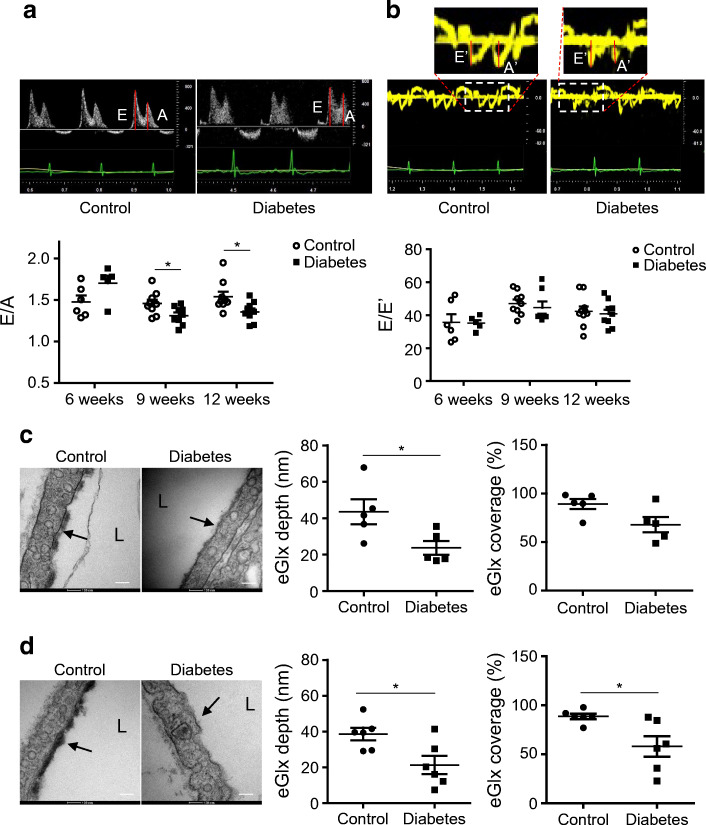
eGlx damage is associated with the development of DCM in a mouse model of type 2 diabetes. The development of DCM was monitored with echocardiography in *db*/*db* mice and control lean mice. (**a**) Representative pulsed wave Doppler images and reduced E/A ratio in diabetic mice when mice were 9 and 12 weeks old, compared with control lean mice (*n* = 6–9 for control mice and 5–9 for diabetic mice; *p< 0.05 [unpaired *t* test]). (**b**) Representative tissue Doppler images and no change of E/E′ in diabetic mice (*n* = 6–9 for control mice and 5–9 for diabetic mice). (**c**, **d**) At the age of 7 weeks (pre-DCM) (**c**) and 12 weeks (after the development of DCM) (**d**) control and diabetic mice were perfusion-fixed with Alcian Blue and glutaraldehyde solution for electron microscopy preparation. High-magnification electron micrographs of capillaries from left ventricles are presented. Scale bar, 100 nm. Arrows point to the eGlx on top of endothelial cells. Significantly reduced eGlx depth in heart capillaries was observed in 7-week-old diabetic mice (**c**; *n* = 5; *p< 0.05 [unpaired *t* test]). Significantly reduced eGlx depth and coverage (% of grid points with eGlx depth > 10 nm) were observed in heart capillaries from 12-week-old diabetic mice (**d**; *n* = 6; *p< 0.05 [unpaired *t* test]). Data are presented as mean ± SEM. L, capillary lumen

Next, we used *db*/*db* and lean mice to investigate the relationship between CMVEC glycocalyx damage and development of cardiac dysfunction in type 2 diabetes. The *db*/*db* mice developed diastolic dysfunction from 9 weeks of age (Fig. 4a, Table 4), as per our previous findings [6], with no change in E/A ratio at 6 weeks and a significant decrease in E/A ratio at 9 and 12 weeks. In comparison with STZ-induced diabetes, no significant change in E/E′ was observed in *db*/*db* mice (Fig. 4b, Table 4). Consistent with the type 1 diabetes model, there was no change in systolic function in *db*/*db* mice up to 12 weeks (ESM Fig. 7). Therefore, mice were further studied at 7 weeks of age when hyperglycaemia but not cardiomyopathy was established (Table 5) and also at 12 weeks. Seven-week-old *db*/*db* mice had significantly reduced eGlx depth and showed a non-significant reduction (*p* = 0.053) in eGlx coverage when compared with lean non-diabetic control mice (Fig. 4c). In 12-week-old *db*/*db* mice, both eGlx depth and coverage were significantly reduced (Fig. 4d). The existence of eGlx damage in both 7-week-old (pre-cardiomyopathy) and 12-week-old (with cardiomyopathy) *db*/*db* mice indicates eGlx damage precedes the development of detectable cardiomyopathy. Interestingly, the lean non-diabetic control mice of this strain appear to have a thicker CMVEC glycocalyx than non-diabetic FVB control mice.

**Table 4:** Pulsed wave and tissue Doppler parameters in *db*/*db* and control lean mice

Age	Pulsed wave Doppler			Tissue Doppler		
	E (mm/s)	A (mm/s)	IVRT (ms)	E’ (mm/s)	A’ (mm/s)	E’/A’
6 weeks						
Control (*n* = 6)	742 ± 19	509 ± 26	21.4 ± 0.8	22.7 ± 2.9	16.2 ± 1.0	1.38 ± 0.13
Diabetes (*n* = 5)	795 ± 44	468 ± 10	21.6 ± 1.5	22.7 ± 1.2	13.2 ± 0.7*	1.73 ± 0.11
9 weeks						
Control (*n* = 9)	721 ± 23	507 ± 23	24.8 ± 0.7	15.4 ± 0.8	20.2 ± 0.7	0.78 ± 0.07
Diabetes (*n* = 9)	652 ± 29	500 ± 24	26.7 ± 2.4	14.0 ± 1.8	17.4 ± 2.2	0.83 ± 0.08
12 weeks						
Control (*n* = 9)	737 ± 28	496 ± 25	24.6 ± 1.1	18.2 ± 1.5	21.4 ± 0.8	0.88 ± 0.05
Diabetes (*n* = 9)	717 ± 17	530 ± 11	23.6 ± 1.1	18.3 ± 1.4	15.8 ± 1.0***	1.17 ± 0.12*

Data are presented as mean ± SEM

*p< 0.05 and ***p< 0.001 vs control

IVRT, isovolumic relaxation time

**Table 5:** Body weight and tail-vein blood glucose levels of *db*/*db* mice

Mouse	5 weeks		6 weeks		7 weeks		12 weeks	
	BW (g)	Glu (mmol/l)	BW (g)	Glu (mmol/l)	BW (g)	Glu (mmol/l)	BW (g)	Glu (mmol/l)
Lean (*n* = 10)	NT	NT	19.15 ± 0.25	NT	21.23 ± 0.33	10.13 ± 0.68	NT	10.65 ± 0.81
*db*/*db* (*n* = 10)	NT	10.15 ± 0.45	20.45 ± 0.59	14.58 ± 1.39	27.23 ± 0.63***	23.87 ± 2.20***	NT	31.17 ± 1.29***

Data are presented as mean ± SEM

***p< 0.001 vs lean control

BW, body weight; Glu, blood glucose; NT, not tested

### Enzymatic eGlx disruption causes ventricular dysfunction in isolated rat hearts

**Fig. 5:**
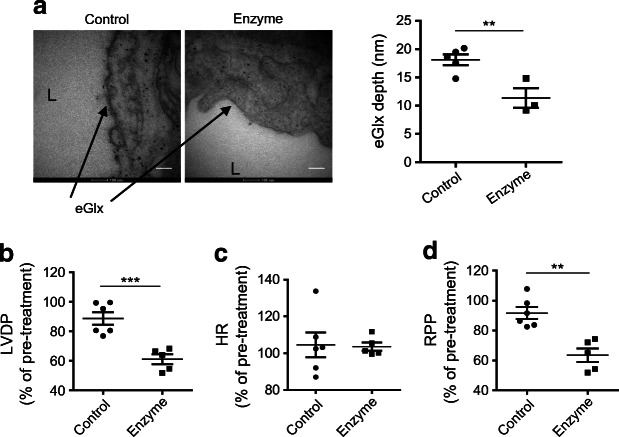
Damage of eGlx with enzyme treatment leads to impaired contractility in rat hearts. Hearts isolated from Sprague Dawley rats were perfused retrogradely with Krebs solution using Langendorff preparation. Once heart beats were stable, the hearts were further perfused with or without the combination of hyaluronidase (14 μg/ml) and chondroitinase (0.0022 U/ml) in Krebs solution for 40 min. (**a**) Damage to the eGlx after enzyme perfusion was identified in hearts perfusion-fixed with Alcian Blue and glutaraldehyde solution for electron microscopy preparation after heart functions were monitored. eGlx depth was decreased in the left ventricle capillaries of isolated rat hearts after enzyme perfusion (*n* = 5 for control and 3 for enzyme-perfused hearts; **p< 0.01 [unpaired *t* test]). Scale bar, 100 nm. (**b**–**d**) Perfusion with the combination of enzymes caused reduced LVDP (***p< 0.001 [unpaired *t* test]) (**b**), left heart rate unchanged (**c**) and reduced rate pressure product (**d**), which is LVDP × HR (**p< 0.01 [unpaired *t* test]). *n* = 5 or 6. Data are presented as mean ± SEM. HR, heart rate; L, capillary lumen; LVDP, left ventricular developed pressure; RPP, rate pressure product

Having demonstrated CMVEC glycocalyx damage in diabetes and its association with cardiac dysfunction in mice, we sought to confirm directly that CMVEC glycocalyx damage causes cardiac dysfunction by using explanted hearts in a Langendorff preparation, thus avoiding confounding haemodynamic factors. Rats were used as the larger heart size improves success rates and reproducibility. Enzymatic removal of CMVEC glycocalyx in Sprague Dawley rat hearts reduced eGlx depth by approximately 40% (Fig. 5a). Myocardial contractility was compromised following this treatment, with a reduction in left ventricular developed pressure when standardised against pre-treatment values (Fig. 5b). The heart rate was not altered, resulting in significant reduction in rate pressure product (Fig. 5c,d).

### Ang1 restores eGlx and improves diastolic heart function in diabetic FVB mice

**Fig. 6:**
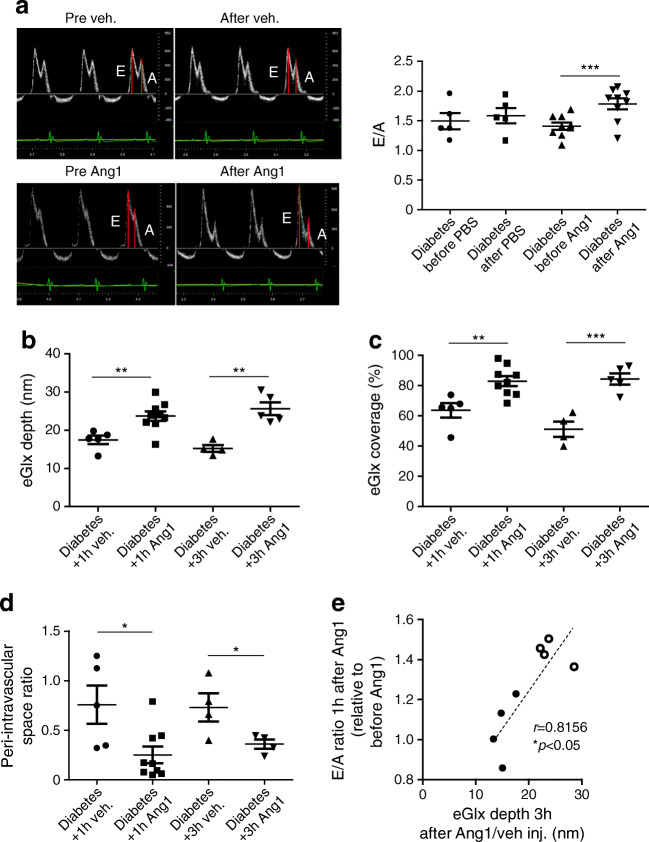
Ang1 improves diastolic heart function of diabetic FVB mice, associated with increased eGlx depth and coverage, and reduces perivascular space. Diabetes was induced in male FVB mice by STZ injections. Nine weeks after STZ injections, one group of diabetic mice were treated with Ang1 (200 ng/ml of blood volume, i.v.). Diastolic function was monitored before and 1 h after treatment. These mice were perfusion-fixed with Alcian Blue and glutaraldehyde solution for electron microscopy preparation 3 h after injection with Ang1. The effects of 1 h treatment with Ang1 or vehicle on eGlx were identified using another batch of mice. (**a**) One hour of Ang1 treatment improves diastolic heart function (*n* = 5 for diabetes+vehicle and 9 for diabetes+Ang1; ***p< 0.001 [unpaired *t* test]). (**b**, **c**) Electron microscopy shows increased eGlx depth at both 1 h (*n* = 5 for diabetes+vehicle and 9 for diabetes+Ang1; **p< 0.01 [unpaired *t* test]) and 3 h after Ang1 treatment (*n* = 4 for diabetes+vehicle and 5 for diabetes+Ang1; **p< 0.01 [unpaired *t* test]) (**b**), and increased eGlx coverage (**p< 0.01 and ***p< 0.001 [unpaired *t* test]) (**c**). (**d**) The enlarged perivascular space in mice with DCM is also reduced by Ang1 (*n* = 5 for diabetes+vehicle and 9 for diabetes+Ang1 for 1 h; *n* = 4 for diabetes+vehicle and diabetes+Ang1 for 3 h; *p< 0.05 [unpaired *t* test]). (**e**) The improvement of heart diastolic function in FVB mice by Ang1/vehicle is correlated with their corresponding eGlx depth identified 3 h after Ang1/vehicle treatments. Black circles, vehicle-treatment; white circles, Ang1 treatment (*n* = 8). Data are presented as mean ± SEM. Inj., injection; veh, vehicle

We next sought to confirm that restoration of the eGlx restores cardiac function in diabetes by administering Ang1 in a mouse model of type 1 diabetes. Since the E/A ratio was consistently reduced in mouse models of both type 1 and 2 diabetes in the present study, we focused on this parameter. Echocardiography was performed before and 1 h after Ang1 treatment. Mice were then perfusion-fixed (3 h after Ang1 injection) for electron microscopy for eGlx measurement. Ang1 improved diastolic heart function measured by E/A ratio in diabetic mice treated with Ang1 (Fig. 6a). CMVEC glycocalyx depth and coverage was increased (Fig. 6b,c) and we found a corresponding reduction in the perivascular space (Fig. 6d), suggesting reduced oedema due to reduced microvascular permeability in Ang1-treated mice. In another experiment we collected tissue for electron microscopy 1 h after Ang1 treatment (at the same time point as the echocardiography) and confirmed that eGlx and perivascular space changes were established at that time point (Fig. 6b–d). There was a significant correlation between the increase in E/A ratio 1 h after Ang1 and the eGlx depth 3 h after Ang1, in the same mice, confirming that Ang1-induced improvement in diastolic function is associated with eGlx recovery (Fig. 6e).

## Discussion

We first showed that CMVEC glycocalyx damage occurs in a model of type 1 diabetes and is associated with diastolic dysfunction. Given that oedema is known to compromise cardiac function [10], we reasoned that eGlx damage may contribute to cardiac dysfunction through increased microvascular permeability. Previous studies in other models have shown that glycocalyx damage increases perivascular space in the left ventricle [28, 35, 36], consistent with an increase in microvascular permeability. Similarly, we observed an increase in perivascular space that was not due to perivascular fibrosis. We then provided in vitro evidence that eGlx disruption is sufficient to increase permeability across CMVEC monolayers.

To investigate potential mechanisms underlying eGlx damage in diabetes, we studied CMVECs isolated from mice in a model of type 1 diabetes. Our findings suggested an increase in MMP expression and a significant increase in active MMP9 was confirmed in myocardial lysates. MMP9 has previously been found to be increased in diabetic kidneys and an MMP2 and MMP9 inhibitor mitigates eGlx damage and reduces albuminuria in that model [37]. Therefore, MMP9 may contribute to the eGlx damage in DCM. As SDC4 is known to be cleaved by MMP9 [20, 37], the loss we observed of this key eGlx component from the myocardial capillaries is consistent with this potential mechanism and further characterises eGlx damage in DCM.

We used *db*/*db* mice to investigate whether eGlx disruption also occurred in type 2 diabetes. We confirmed eGlx damage in this model and showed that CMVEC glycocalyx damage preceded diastolic dysfunction, consistent with eGlx damage having a causative role in cardiac dysfunction.

We confirmed the importance of eGlx for heart function by enzymatic stripping of CMVEC glycocalyx in hearts isolated from rats. This procedure resulted in a reduction in contractility, demonstrating that CMVEC glycocalyx damage causes myocardial dysfunction, further supporting the possibility of a causative role for eGlx damage in DCM. We demonstrated systolic dysfunction in isolated rat hearts (diastolic function is not measured in that model) while in our mouse models we detected diastolic dysfunction but not systolic dysfunction. We suggest that this apparent contradiction is resolved by the appreciation that different models present different phenotypes of heart dysfunction rather than different severities.

Ang1 treatment restored eGlx depth and coverage in CMVECs in the type 1 diabetes model along with a reduction in perivascular space and improved diastolic function. The effect of Ang1 on eGlx in 1 h was expected from our previous experience in the mesentery [22] and in diabetic nephropathy in which eGlx is restored by Ang1 within 30 min [21]. Our previous work showed that Ang1 leads to translocation of eGlx components to the cell surface (accounting for the rapid action) and that the ability of Ang1 to restore the microvascular permeability barrier depends on eGlx [22]. It is notable that both the perivascular oedema and the E/A also improved within this short timescale. These observations indicate that in this model, diastolic dysfunction is reversible upon correction of eGlx damage and hence the microvascular permeability defect.

Our findings in this study have several important implications. The reversibility observed in the model of type 1 diabetes suggests that therapies restoring eGlx may improve cardiac function before long-term consequences of increased permeability are established. As the plasma *t*½ of Ang1 is short [38] and parenteral administration is required, Ang1 itself is unlikely to provide a viable therapy. Other endothelial growth factors, including VEGF-C [39] and VEGF-A_165_b [16], can also restore eGlx with functional benefits but have the same limitations. However, novel strategies to target the Tie2 receptor are in development [40], as are other approaches to protect and restore the eGlx [11], and have potential for benefit in human disease. eGlx damage has been observed in other circulations in diabetes suggesting that eGlx damage may be a common pathway for the progression of chronic diabetic microvascular complications, including cardiomyopathy, nephropathy [16, 41] and retinopathy [42]. Therefore therapies targeting the eGlx may simultaneously protect against multiple complications.

Our work on eGlx dysfunction in DCM contributes to a wider understanding of microvascular pathology. Acute and chronic coronary microvascular permeability changes have been reported in hypertension, hypoproteinaemia and in both experimental and clinical sepsis [10, 43], and are a major contributor to endotoxin-induced myocardial dysfunction [44]. The association of sepsis-induced myocardial oedema, with a loss of negatively charged endothelial components, also links the eGlx to increased microvascular permeability [45].

The development of albuminuria in our type 1 diabetes model (ESM Fig. 8), in addition to DCM, is consistent with other evidence linking loss of eGlx, albuminuria and vascular dysfunction [46] and suggests that eGlx damage may explain the association of human DCM with microalbuminuria [4, 5, 47]. Hence, microalbuminuria in diabetes may help to identify those individuals who should be screened for DCM and/or who may benefit from eGlx-targeted therapies.

In this study we have concentrated on the role of CMVEC glycocalyx in the permeability barrier. However, other consequences of eGlx dysfunction may also contribute to the development of DCM. For example, eGlx damage has been associated with disturbed microvascular flow [48], which reduces myocardial oxygen delivery and metabolite clearance. eGlx damage may also interfere with nitric oxide signalling and promote interactions of platelets and leucocytes with the endothelium.

We developed a novel lectin-based fluorescence imaging technique to characterise the eGlx and validated it in a mouse model of type 1 diabetes by comparison with electron microscopy, the ‘gold standard’ technique for measuring eGlx. We then used this technique in human samples to confirm its broader applicability. This technique renders immersion-fixed tissue, including human tissues, suitable for eGlx analysis and hence will accelerate the field of eGlx research.

Our study has some limitations. We recognise that the mouse models used do not fully recapitulate human disease but the consistency of the eGlx damage and diastolic dysfunction in the two models suggests that our results are generalisable. For diastolic dysfunction analysis, increase in E/E′ has been used in addition to reduced E/A ratio [27, 49] and so we explored the use of E/E′ in our study. We saw increased E/E′ in our model of type 1 diabetes wherein the change was more marked than the E/A ratio. However, we did not see change in E/E′ in the model of type 2 diabetes (*db*/*db* mice), although the reduction in E/A ratio was obvious. A limitation of E/A and E/E′ analysis is that they are load-dependent: both preload and afterload affect these measures of diastolic function. Therefore, we also used the ex vivo Langendorff preparation in which contractile function can be assessed in isolation from confounding haemodynamic factors. However, the lack of load-independent measures in a diabetes model is a limitation of our study.

### Conclusions

Our results identify a correlation between loss of eGlx and the development of DCM. Moreover, loss of eGlx is likely a contributing factor towards the progress to DCM from hyperglycaemia, suggesting that eGlx could be a therapeutic target for individuals with DCM. We have found that short-term treatment with Ang1 improved heart function in diabetic mice, in parallel with its restorative effects on diabetes-induced eGlx damage and oedema. The effect of long-acting Tie2 agonists on eGlx protection and on DCM progression should be investigated to establish whether the eGlx restoration and improvement in diastolic function can be sustained. Our data suggest that targeting increased MMP activity could be an alternative approach.

## Supplementary Information

ESM 1 (PDF 1.08 MB)

## Data Availability

The authors declare that all data supporting the findings of this study are available within the article and its supplementary information files.

## Abbreviations

Ang1: Angiopoietin 1
ciCMVEC: Conditionally immortalised CMVEC
CMVEC: Coronary microvascular endothelial cell
DCM: Diabetic cardiomyopathy
eGlx: Endothelial glycocalyx
FACS: Fluorescence activated cell sorting
GAG: Glycosaminoglycan
IB4: Isolectin B4
LEA: *Lycopersicon esculentum* agglutinin
MAL I: *Maackia amurensis* lectin I
MMP: Matrix metalloproteinase
MOA: *Marasmium oreades* agglutinin
PFA: Paraformaldehyde
qRT-PCR: Quantitative reverse transcription-PCR
SDC4: Syndecan 4
SNA: *Sambucus nigra* agglutinin
STZ: Streptozotocin
TEM: Transmission electron microscopy
tsSV40LT: Temperature-sensitive simian virus 40 large tumour
VE-cadherin: Vascular endothelial cadherin
VEGF: Vascular endothelial growth factor
VEGFR2: Vascular endothelial growth factor receptor 2
WGA: Wheatgerm agglutinin
