# Supplementary material for: Endothelial glycocalyx is damaged in diabetic cardiomyopathy: angiopoietin 1 restores glycocalyx and improves diastolic function in mice
Source: Diabetologia. 2022 Feb 25;65(5):879–94. doi: 10.1007/s00125-022-05650-4 (PMC8960650; doi:10.1007/s00125-022-05650-4)
Supplement: Supplementary file 1 — (PDF 1.08 MB) [file 125_2022_5650_MOESM1_ESM.pdf]

SUPPLEMENTARY INFORMATION

Detailed methods

Cell culture and treatment

Primary human CMVECs (CC-7030, Lonza, Basel, Switzerland) were cultured in endothelial growth medium 2 microvascular (EGM2-MV; Lonza, Basel, Switzerland) containing 5% vol./vol. FCS and growth factors as supplied with the exception of vascular endothelial growth factor (VEGF), at 37°C. Human conditionally immortalised CMVECs (ciCMVECs) were generated and have been characterised (ESM Fig. 1). Briefly, primary human CMVEC were transduced with temperature-sensitive simian virus 40 large tumour (tsSV40LT) antigen and telomerase using retroviral vectors. At the permissive temperature of 33°C, the tsSV40LT transgene is activated, causing cell proliferation (without telomere shortening), whereas at 37°C, the transgene is inactivated, rendering cells non-proliferative and quiescent. The ciCMVECs were cultured in the same medium as for primary CMVECs and used for experiments after 5 days at the non-permissive temperature. Mycoplasma contamination is regularly checked for ciCMVEC and no contamination has been found. The primary CMVEC was achieved commercially, and the report on mycoplasma contamination status of this cell line is negative.

Protein extraction and Western blotting

To extract protein, the monolayer of primary CMVECs, undifferentiated ciCMVECs and differentiated ciCMVECs were lysed with 200 µl of ice-cold lysis buffer (RIPA buffer was from Thermo Fisher Scientific, UK. Both protease inhibitor and phosphatase inhibitor were from Merck Life Science, Gillingham, UK) for 30 min. The monolayer was subsequently scraped, passed through a 27G needle ten times, and spun at 13000 *g* for 30 min at 4°C. The supernatant containing the protein was then used for western blotting.

For western blotting, lysate of primary CMVECs was used as control. Protein samples were separated by SDS-PAGE and were blotted onto polyvinylidene fluoride (Immobilon-P; Millipore Corp., Billerica, MA) membrane. After blocking in 5% wt/vol. BSA (Sigma-Aldrich, Dorset, UK) in 0.1% vol./vol. Tween 20 in tris-buffered saline (TBS-T) for 1 hour, the membranes were incubated overnight at 4°C with primary antibodies against the endothelial markers CD31 (Ab28364, Abcam, Cambridge, UK), vascular endothelial cadherin (VE-cadherin, sc – 9989; Santa Cruz Biotechnology, Heidelberg, Germany), VEGF receptor 2 (VEGFR2, 05-554; Merck Life Science, Gillingham, UK) and β-actin (Merck Life Science, Gillingham, UK) at 1:1000 in 3% BSA in 0.1% TBS-T. After being washed thoroughly in 0.1% TBS-T, the membranes were incubated with corresponding secondary antibodies in 3% BSA in 0.1% TBS-T before being washed and developed in ECL.

Animal models of diabetes

Experiments were performed in accordance with the Guide for the care and use of laboratory animals, Eighth edition (2011). All animal procedures performed conformed to the guidelines from Directive 2010/63/EU of the European Parliament on the protection of animals used for scientific purposes and were approved by the University of Bristol and the British Home Office (Licenses: PPL 30/2811 and PPL 30/3373).

Type 1 diabetes was induced in male FVB mice (TG 287 SATOJ; The Jackson Laboratory, USA) by injection of streptozotocin (STZ; 50 mg/kg body weight per day, i.p. for 5 consecutive days) [1]. Control mice received citric acid buffer. All mice were fasted for 4–6 h before administration of STZ. Hyperglycemia was confirmed by blood glucose levels of > 16 mmol/L on two consecutive days. Only mice remaining hyperglycemic over the duration of the study were included. Body weight and blood glucose were monitored weekly with a glucose meter (ACCU-CHEK Aviva; Roche, UK) from 1 week after administration of STZ until the end of the study (9 weeks after STZ injection).

In addition, male leptin-receptor mutant *db/db* mice (BKS.Cg-+*Lepr^{db}*/*Lepr^{db}*/OlaHsd; Harlan, UK) were used as a model of insulin-resistant type 2 diabetes. Increases of blood glucose begin at 6 weeks of age and diastolic dysfunction from 9 weeks in these mutant mice [1]. Age-matched lean mice (BKS.Cg-*m+/+Lepr^{db}*/OlaHsd) were used as control. Two groups of mutant mice were studied in this project, one at early diabetes (7 weeks old) and the other at a later (12 weeks old) time point. Hyperglycemia was assessed as above.

Hemodynamic measurements

Diastolic dysfunction is the major phenotype in the development of DCM [2]. Diastolic function was determined by both E/A ratio with pulsed wave Doppler [1, 3] and E/E' with tissue Doppler [4] using a high-frequency, high-resolution echocardiography system (Vevo 3100; VisualSonics, Toronto, Canada). Images were captured at a heart rate of 380 ± 10 beats/ min under 1–3% vol./vol. isoflurane anaesthesia. Systolic function was determined with the analysis of M-mode images at a heart rate of 450 ± 30 beats/ min. Mice were excluded from the experiments if their heart rates did not reach the optimal range for the measurement of either diastolic or systolic function within 30 min after initiating monitoring.

Determination of eGlx depth and coverage, perivascular space and endothelial cell thickness

To quantify eGlx parameters by transmission electron microscopy (TEM), mouse hearts were stopped at the end of diastole by the injection of 2.5 ml of 0.1 mol/L $CdCl_2$ and perfusion-fixed for electron microscopypreparation as previously described [5]. To study CMVEC glycocalyx, four capillaries were analysed at random from each left ventricle section (one per mouse). TEM images with nominal 49,000x magnification (Pixel resolution) were acquired from opposite sides of each capillary wall where a clear phospholipid bilayer was visible. Images were blinded, and measurements were taken randomly using a predefined algorithm using ImageJ as previously [5]. A fixed digital grid with 14 lines was superimposed over the electron micrograph, eGlx depth was measured at each cross point between grid and endothelial cell membrane. eGlx coverage was defined as the proportion of points where eGlx depth was 10 nm or greater. Approximately 80 measurements of eGlx depth were made on each heart section.

Perivascular space area was determined using Image J and presented as a ratio relative to the area inside the capillary [6]. The thickness of the capillary endothelium was determined as follows. Two cross lines were applied at the center of an image with Image J. The thickness at the 4 points where the 2 central lines intersect with the capillary wall was measured as the shortest distance between the inner and outer endothelial plasma membrane.

Trichrome staining to identify perivascular fibrosis

To investigate whether fibrosis formation accounts for the increased perivascular space in diabetic FVB mice, mouse heart sections were stained with Trichrome Stain (Masson) Kit (Sigma-Aldrich, UK) according to the manufacturers' instructions.

Localising lectin binding molecules using correlative light and electron microscopy in mouse and human tissues

Until now, eGlx labelling has relied on perfusion fixation techniques which are technically demanding, require the use of a whole animal and are limited to animal samples. We developed a novel technique to localise lectin-binding to the eGlx in immersion-fixed samples using correlative light and electron microscopy. Importantly our novel technique renders immersion-fixed tissue, including human tissues, suitable for eGlx analysis and hence will accelerate the field of eGlx research.

Mice were perfusion-fixed with 4% wt/vol. paraformaldehyde (PFA) in PBS, and hearts were dissected and embedded in paraffin. Five micrometers cut sections were mounted onto glass slides and left to dry overnight at 37°C. Sections of normal human myocardium were obtained from the Bristol Coronary Biobank (ethical approval 08/H107/48). For lectins staining, sections were dewaxed and rehydrated, blocked with 1% BSA in PBS containing 0.5% vol./vol. Triton-X (PBX). Specific endogenous biotin blocking (Vector Laboratories, UK) was then applied. Sections were incubated with biotinylated *Maackia amurensis* lectin I (MAL I, B-1315, from Vector Laboratories, UK), *Marasmium oreades* Agglutinin lectin (MOA, BA-9001-1, from EY Laboratories, UK), *Sambucus nigra* lectin (SNA, B-1305, from Vector Laboratories), *Lycopersicon esculentum* (LEA, B-1175, from Sigma Aldrich, UK), *Isolectin* B4 (IB4, I21414, from Vector Laboratories) at 20 µg/ml and biotinylated Wheat germ agglutinin (WGA, B-1025, from Sigma Aldrich) at 25 µg/ml in 1% BSA in PBX, pH 6.8, overnight at 4°C. After washing, 2 nmol/l quantum dots 655 (6x12 nm)-conjugated streptavidin (Q10123MP, Thermo Fisher Scientific, UK) in 1% BSA in PBX, pH 6.8 was applied for 1 h at room temperature. Fluorescence images were captured to confirm specific staining. Sections were then post-fixed in OsO_4 for electron microscopy imaging after being post-fixed for 15 min in 2.5% vol./vol. glutaraldehyde (Elektron Technology, UK) in 0.1 mol/l phosphate buffer, pH 7.4. 3% wt/vol. uranyl acetate in water was applied, followed by graded ethanol dehydration, infiltration with Epon (TAAB Labs Ltd, Aldermaston, UK) and polymerization at 60°C. The sections were stripped off the glass slide by sticking a resin block on the resin embedded sections and then repeated freezing in liquid nitrogen and thawing in boiling water to break the resin away from the glass. The tissue in the resin block was finally sectioned at 70 nm thickness before images were taken on an FEI Tecnai 12 (120KV BioTwin Spirit) transmission electron microscope (FEI, Cambridge, UK).

Lectin based fluorescence and immunofluorescence to quantify eGlx and its component

Tissue sections were prepared as above. After sections were incubated with biotinylated lectins and washed, 2 µg/ml of Alexa Fluor 488-conjugated streptavidin (Vector Laboratories, UK) in 1% BSA in PBX, pH 6.8, was applied for 1 h at room temperature. DAPI (Thermo Fisher Scientific, UK; 10 µg/ml in 1% BSA in PBX, for 5 min) was applied for counterstaining. Images were acquired with a Leica SP5-II confocal microscope (Leica Microsystems, UK) and lectin fluorescence intensity was normalized to that of DAPI.

For SDC4 expression, 5 µm cut frozen heart sections were fixed with 4% PFA for 15 min. The sections were incubated with primary antibodies (purified rat-anti-mouse SDC4, Clone KY/8.2 from BD Biosciences, UK and VE-cadherin, SC-9989, mouse-anti-mouse, from Santa Cruz Biotechnology, Heidelberg, Germany, at a 1:50 dilution) in 1% BSA overnight at 4°C after blocking. After washing, sections were incubated with 2 µg/ml of Alexa Fluor™ 488 anti-rat and Alexa Fluor™ 546 anti-mouse secondary antibodies (Thermo Fisher Scientific, UK), followed with counterstaining with DAPI. Images were captured using a Leica SP5-II

confocal laser scanning microscope and analysed with Coloc 2 in Fiji. Colocalisaton was presented as the proportion of the VE-cadherin-stained area that also had SDC4 staining.

Measurement of transendothelial protein passage

Twelve-millimeter transwell (0.4 µm pore polyester membrane insert, 1.12 cm^2 surface area; Corning, UK) were seeded with ciCMVECs at 37,500 cells/cm^2. Inserts were placed in 12-well plates. Cells were cultured at 33°C for 2 days and 37°C for 5 days. Media were changed three times a week. Transendothelial permeability to macromolecules was assessed by measurement of passage of Alexa Fluor 488-conjugated BSA (Thermo Fisher Scientific, UK) across the monolayer in tissue culture inserts. The cells were starved of serum for 2 h and then treated with a combination of enzymes (heparinase 1 U/ml + hyaluronidase 4.5 U/ml + chondroitinase 100 mU/ml) for 3 h before protein passage measurement. Fifty microliters of media from inserts and wells were replaced with 1 mg/ml AF488-conjugated BSA and unconjugated BSA respectively. At 1 and 2 h, 100 µl aliquots were removed and replaced with 100 µl of media containing unconjugated BSA (100 µg/ml). The fluorescence of the aliquots was measured, and the amount of AF488-BSA passing through the monolayer was calculated by reference to a set of BSA standard dilutions.

Fluorescence labelling

To confirm eGlx removal, ciCMVECs were fixed and stained with FITC-labelled wheatgerm agglutinin (L4895, Sigma, UK), as previously described [7]. To confirm that the cell monolayer remained intact after enzyme treatment, cells were stained with anti-VE-cadherin as previously [8].

Staining for the expression of VE-cadherin, an endothelial cell junctional marker, was carried out to show the intactness of cells under treatment. In brief, confluent ciCMVECs cultured on coverslips were fixed with 4% PFA for 15 min. Cells were then incubated in blocking solution (5% vol./vol. normal goat serum in PBS containing 0.3% Triton X) for 1 h, and with primary antibodies (VE-cadherin, SC-9989, mouse-anti-mouse, from Santa Cruz Biotechnology, Heidelberg, Germany, at a 1:50 dilution) in 1% BSA overnight at 4°C. After washing, sections were incubated with 2 µg/ml of Alexa Fluor™ 546 anti-mouse secondary antibody (Thermo Fisher Scientific, UK). After 3 washes, the nuclei were counterstained with DAPI. After wash in PBS, sections were mounted in Vectashield mounting medium (Vector Laboratories, Peterborough, UK) and examined using a Leica SP5-II confocal laser scanning microscope attached to a Leica DMI 6000 (Leica Microsystems, UK) inverted epifluorescence microscope.

Fluorescence activated cell sorting

To identify the genes relevant to eGlx synthesis and shedding, heart endothelial cells were collected by fluorescence activated cell sorting (FACS) from both control and diabetic FVB mice 9 weeks after STZ injection. Briefly, left ventricle was digested with 1 mg/ml collagenases (I, IV, V) (Sigma-Aldrich, Dorset, UK) for 1 h at 37°C in a hybridization oven with constant rotation. After the enzymatic digestion was complete, the digest was passed through a 35 µm mesh cell strainer (BD falcon 352235, Thermo Fisher Scientific, UK) to give a single-cell suspension. After 3 washes, the cell pellet was resuspended in HBSS containing 1 mM EDTA, 0.1 µl/ml DNAse and 0.3% BSA and immunostained with phycoerythrin rat-anti-mouse CD31 antibody (550350; BD Pharmingen, San Jose, USA) at a 1:50 dilution for 1 h at 4°C. After 3 washes, the cells were stained with propidium iodide to exclude dead cells and FACS was carried out on the live cells. Controls included unstained cells and isotype control (rat IgG2a, κ isotype control, FITC goat anti-rat Igs; BD Pharmingen, USA) were used for setting up the gates.

TaqMan qRT-PCR array

Total RNA was extracted from heart endothelial cells collected by FACS from control and diabetic FVB mice 9 weeks after STZ injection. The custom-designed TaqMan Array Card (Applied Biosystems) is a 384-well microfluidic card that performs 384 simultaneous real-time qRT-PCR. We designed an array including 96 glycocalyx-related and endothelial genes [9]. cDNA (1:5 dilution) and TaqMan gene expression master mix (Applied Biosystems) were used to perform the reaction. In brief, the wells of the TaqMan Array contained TaqMan gene expression assays that detected the real-time amplification of the specified targets. Relative levels of gene expression were determined from the fluorescence data generated during PCR using a ViiA7 Real-Time PCR System (Applied Biosystems). Expression Suite software (Thermo Fisher Scientific, UK) which utilises the comparative C_T ($\Delta\Delta C_T$) method was used to rapidly and accurately quantify relative gene expression across a large number of genes and samples. For the selected genes of interest, the $2^{-\Delta\Delta Ct}$ method was also used to calculate fold changes, normalised to the geometric mean of 18s and β-actin. Independent *t* tests with $p < 0.05$ were used as a screening test.

MMP activity assay

left ventricle was dissected from control and diabetic FVB mice 9 weeks after STZ injection. Total protein was extracted. MMP2 Biotrak Activity Assay (GE Healthcare Life Sciences, Buckinghamshire, UK) and MMP9 ELISA (AnaSpec, Fremont, CA, USA) were carried out according to the manufacturer's instructions. The concentrations of active MMP2 and MMP9 were normalised to total protein.

Langendorff preparation to measure effects of enzymatic eGlx removal

Seven-week-old male Sprague-Dawley rats (approximately 250 g; Harlan, Bicester, UK) were euthanised by cervical dislocation and hearts quickly removed, mounted on a Langendorff apparatus and perfused in a non-recirculating mode with Krebs solution (NaCl 120 mmol/l, KCl 4.8 mmol/l, $MgSO_4$ 1.2 mmol/l, KH_2PO_4 1.17 mmol/l, $NaHCO_3$ 25 mmol/l, $CaCl_2$ 2.0 mmol/l, glucose 11 mmol/l gassed with 95% vol./vol. O_2–5% vol./vol. CO_2, pH 7.4, 37°C) at a constant flow of approximately 11 ml min^{-1} g^{-1} wet weight as previously [10]. Contractile function was measured using a latex balloon in the left ventricle. Data acquisition and analysis used a PowerLab System (AD Instruments, Bella Vista, NSW, Australia). Left ventricular developed pressure was calculated as the difference between left ventricular systolic pressure and left ventricular end-diastolic pressure. Measurement was initiated after hearts were stabilised by perfusion for 30 min. Hearts were then perfused for 40 min with the combination of hyaluronidase (14 μg/ml; Sigma Aldrich, UK) and chondroitinase (0.0022 u/ml; Sigma Aldrich, UK) in Krebs solution to deplete eGlx or with Krebs solution alone as control. Some isolated hearts were also perfusion-fixed with glutaraldehyde and Alcian Blue for analyzing eGlx by electron microscopy as above.

Restoration of eGlx in vivo

Rescue experiments were performed in the FVB mouse model of type 1 diabetes, aiming to restore the eGlx alteration and reverse diastolic dysfunction, by a single injection of Ang1. Nine weeks after STZ injection, FVB mice were randomised to receive 100 μl of Ang1 (i.v. through the retro-orbital vein to achieve 200 ng/ml of blood volume) or vehicle (PBS, 100 μl) and diastolic function was assessed by echocardiography. Other mice received the same treatments and were perfusion-fixed for electron microscopy and eGlx analysis at 1 h or 3 h after Ang1 injection.

Statistical analysis

All statistical analyses were conducted with Prism version 5.00 (GraphPad Software, Inc., USA) (*$p < 0.05$, **$p < 0.01$, ***$p < 0.001$; ns, not significant). All data are expressed as mean ± SEM and all *n* numbers represent biological repeats. A Student's two-tailed *t* test was used to determine the significance of the difference between means of two groups and Pearson *r* test was used to determine how strongly two groups of data related when data passed a normality test. A normal distribution of the data was tested using the Kolmogorov–Smirnov test if the sample size allowed. If normal distribution or equal-variance assumptions were not valid, statistical significance was evaluated using the Mann–Whitney test for two groups or Spearman *r* test for correlation. Mice were randomly assigned to treatment groups. Tested samples were assayed in a blinded fashion.

Supplementary Figures and Figure Legends

ESM Fig. 1

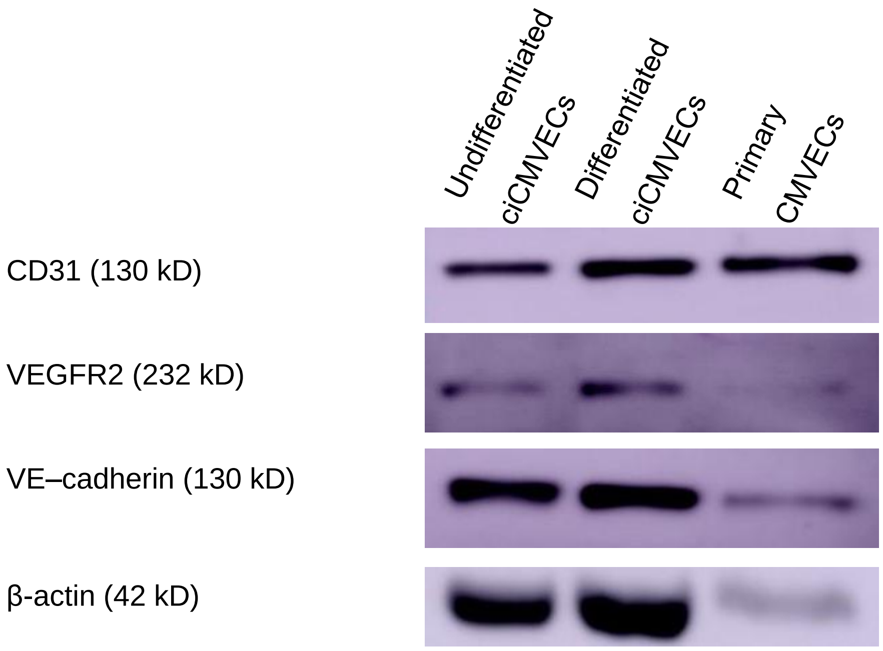

ESM Fig. 1. Characterisation of ciCMVECs by Western blotting.
Primary CMVECs and conditionally immortalised CMVECs (ciCMVECs, both undifferentiated and differentiated) were cultured until cells were approximately 70% confluency. Protein was extracted from cells and subjected to Western blotting. Blots were probed with antibodies against endothelial markers CD31, VEGFR2, VE-cadherin and also β-actin as a loading control. ciCMVECs express typical endothelial markers as expected.

ESM Fig. 2

a

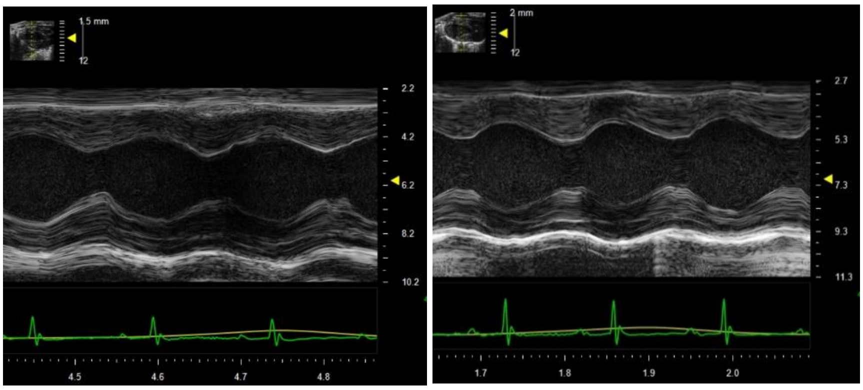

Control Diabetes

b

EF (%)		FS (%)		CO (ml/min)	
Control	Diabetes	Control	Diabetes	Control	Diabetes
75.0±4.2	80.4±3.2	44.6±3.8	49.1±3.4	19.2±0.90	18.5±1.3

ESM Fig. 2. Systolic dysfunction was not detected in diabetic FVB mice. Heart systolic function from control and diabetic mice 9 weeks after STZ injections was monitored with echocardiography. (**a**) Representative M-mode images from control and diabetic mice. (**b**) Results of ejection fraction (EF), fractional shortening (FS) and cardiac output (CO) of the control and diabetic groups.

ESM Fig. 3

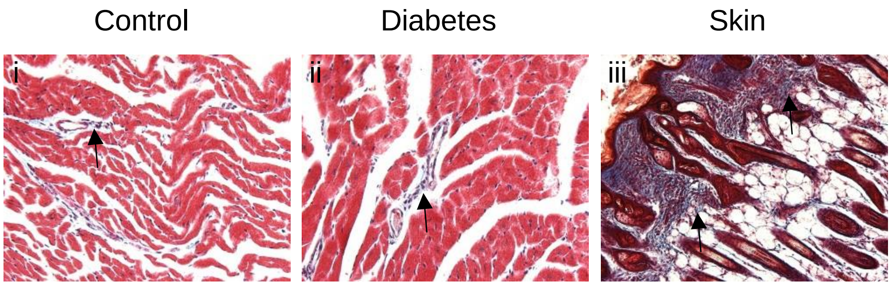

ESM Fig. 3. There is no perivascular fibrosis formation in diabetic FVB mice.
Heart sections from control and diabetic mice 9 weeks after STZ injection were subjected to trichrome staining. Arrows point to collagen stained in blue. There is mild collagen staining in the perivascular space in both control (i) and diabetic (ii) hearts, but with no obvious difference. Skin tissue (iii) shows profound collagen deposition. Scale bar, 10 µm.

ESM Fig. 4

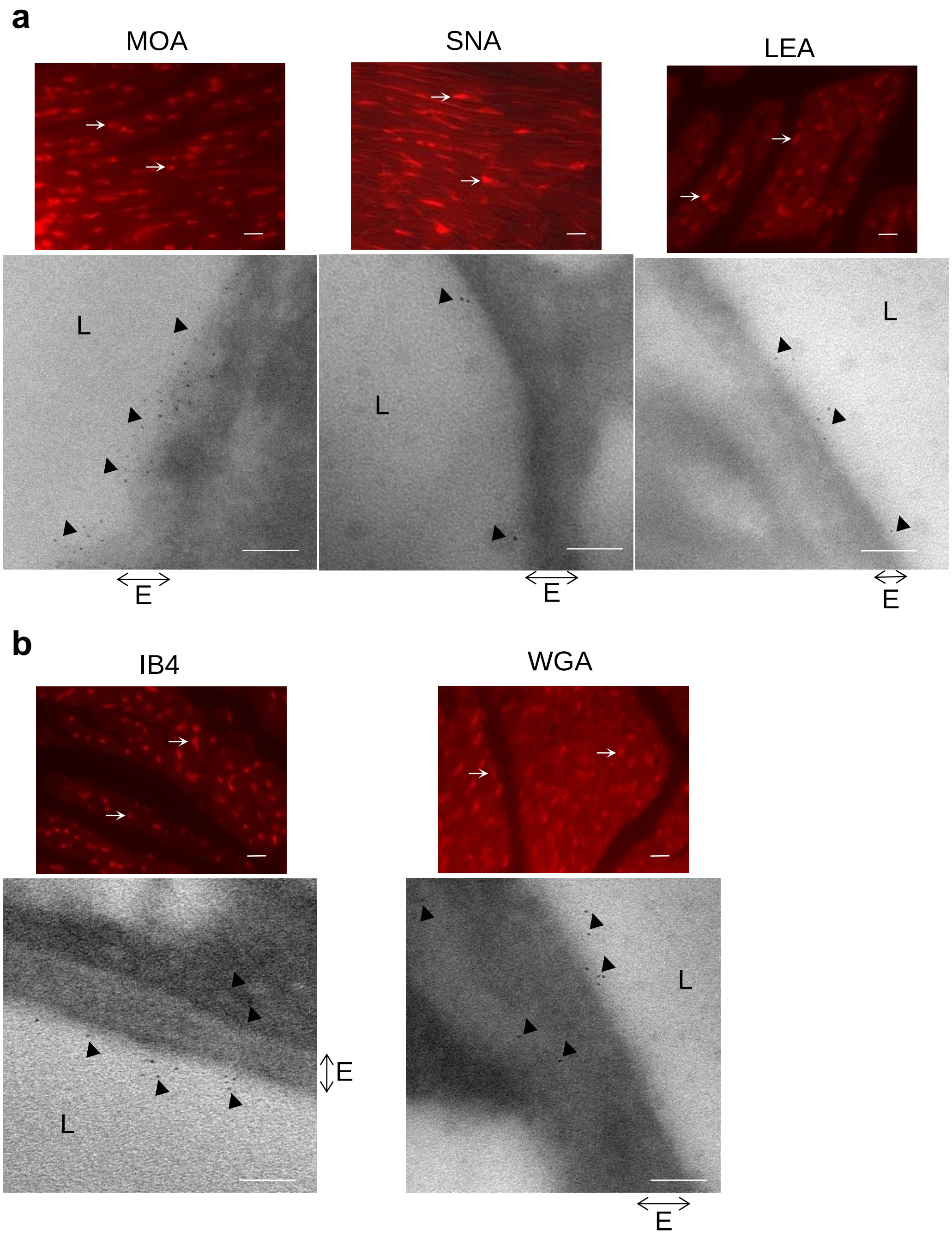

ESM Fig. 4. Localisation of lectin binding molecules in FVB mouse coronary capillaries was determined with correlative light and electron microscopy.
Lectins MOA, SNA, LEA, IB4 & WGA were tested for their binding activities on 4%PFA-fixed, paraffin-embedded FVB mouse heart sections with correlative light and electron microscopy using quantum dots binding to lectins. (**a**) MOA, SNA & LEA lectin binding molecules are mainly expressed in eGlx. SNA binding molecules are sparsely expressed in eGLx. Scale bar, 15 µm in fluorescent images and 100 nm in electron microscopy images. Arrow heads point to quantum dots associated with lectin binding molecules. E, endothelium; L, capillary lumen. (**b**) IB4 & WGA lectin binding molecules are expressed in both eGlx and basement membrane.

ESM Fig. 5

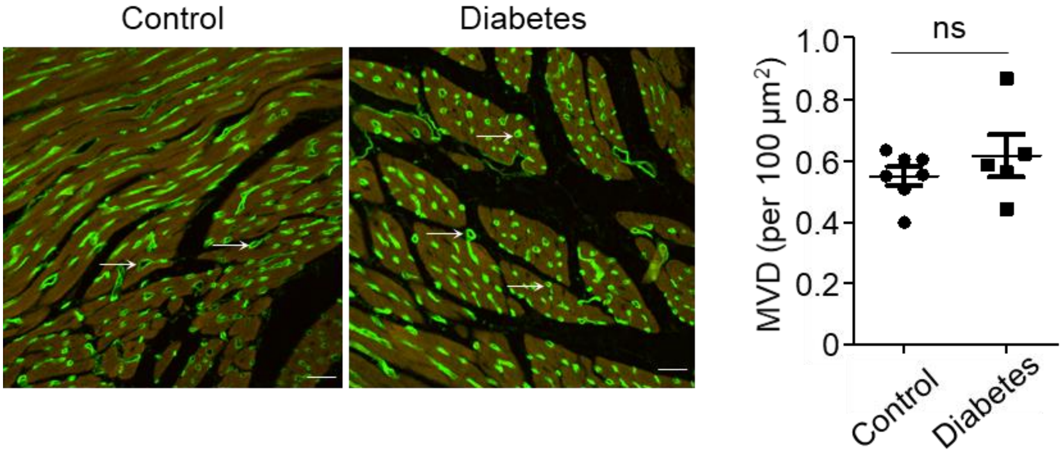

ESM Fig. 5. No difference in the microvascular density (MVD) in left ventricles was observed between control and diabetic mouse hearts.

WGA was used as a marker for microvessels on the basis of WGA expression was confirmed in both eGlx and basement membrane. There is no significant difference between control and diabetic group in the number of microvessels, indicating no difference in microvascular density (n=5–7; ns, not significant [unpaired *t* test]). Arrows point to coronary microvessels stained with WGA. Scale bar, 25 μm.

ESM Fig. 6

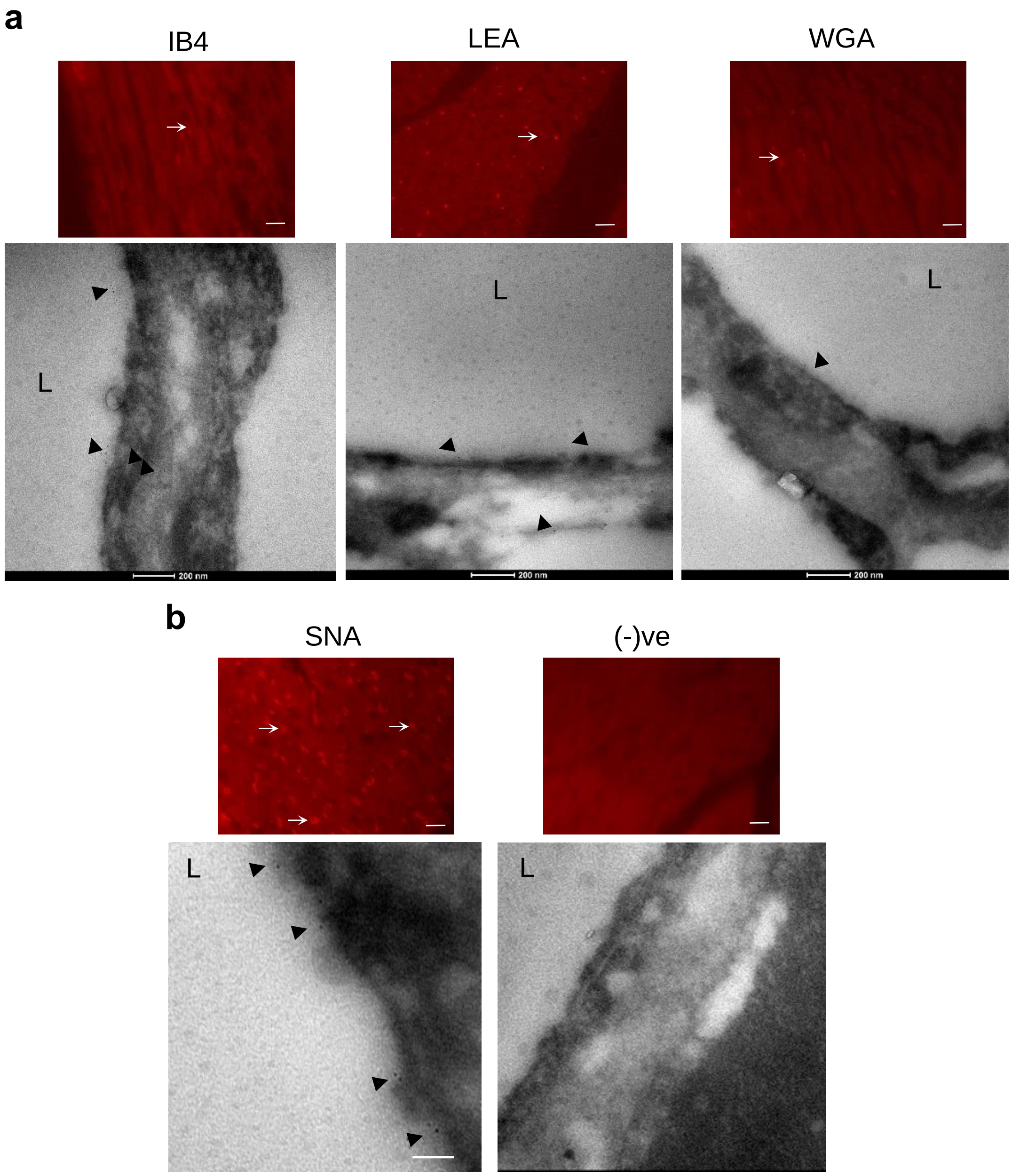

ESM Fig. 6. Lectins bind to eGlx in human coronary capillaries.
Lectins were tested for their binding activities on 4% PFA-fixed, paraffin-embedded human heart sections with correlative light and electron microscopy using quantum dots binding to lectins. (**a**) IB4, LEA & WGA binding molecules are expressed in both eGlx and basement membrane in human coronary microvessels. (**b**) SNA binding molecules are mainly expressed in eGLx. Scale bar, 15 µm in fluorescent images and 200 nm in electron microscopy images. Arrow heads point to quantum dots associated with lectin binding molecules. L, capillary lumen; (-)ve, negative control, in which staining was performed without a lectin incubation.

ESM Fig. 7

a

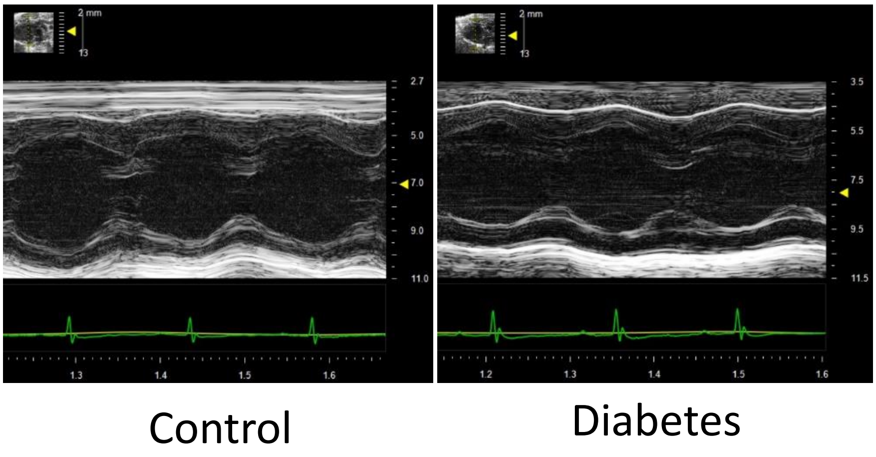

b

Age	EF (%)		FS (%)		CO (ml/min)	
	Control	Diabetes	Control	Diabetes	Control	Diabetes
6 weeks	79.8±4.9	70.2±4.1	49.5±4.6	39.3±3.3	17.6±0.8	15.2±0.6
9 weeks	79.9±3.2	81.3±3.6	49.3±3.6	51.0±4.1	19.5±1.3	19.9±1.8
12 weeks	68.0±5.0	77.1±5.0	38.9±4.4	47.7±4.9	20.1±2.2	17.3±1.9

ESM Fig. 7. No systolic dysfunction was observed in diabetic *db/db* mice at 12 weeks of age.
Heart systolic function was monitored on lean and *db/db* mice with echocardiography. (**a**) Representative M-mode images from control lean and *db/db* diabetic mice when mice were 12 weeks old. (**b**) Results of ejection fraction (EF), fractional shortening (FS) and cardiac output (CO) when the mice were 6, 9 and 12 weeks old. Data are presented as mean ± SEM.

ESM Fig. 8

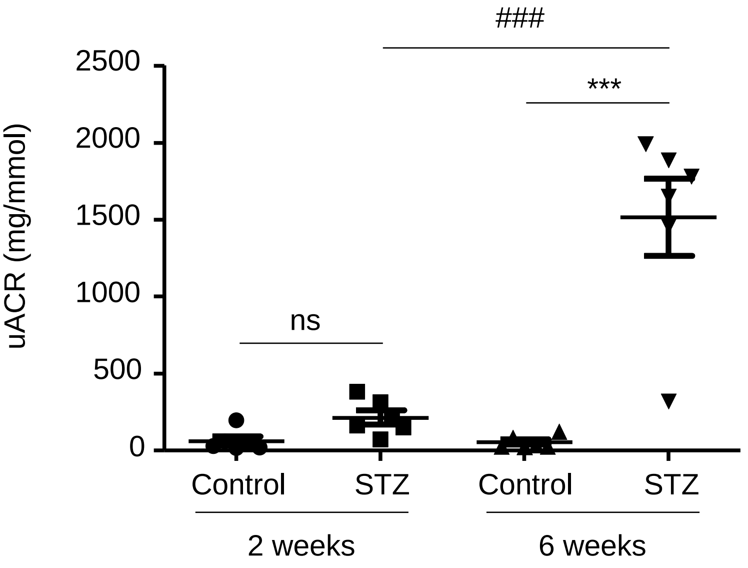

ESM Fig. 8. The type 1 diabetes model in FVB mice were further characterised and FVB mice develops albuminuria over time after STZ injection.
Diabetes was induced in FVB male mice with low doses of STZ injection. Urine was collected at 2 and 6 weeks after STZ injection. Albumin and creatinine levels in the urine were measured. There is no significant increase in urine albumin creatinine ratio (uACR) in diabetic mice 2 weeks after STZ injection. ns, not significant. By 6 weeks after STZ injections, uACR in diabetic mice is 29-fold of that in control mice (***$p<0.001$) and is also dramatically higher than that in 2-week-old diabetic mice ($^{###}p<0.001$ vs 2-week-old STZ [one-way ANOVA with Bonferroni post-hoc tests]).

Supplementary References

[1] Katare RG, Caporali A, Oikawa A, Meloni M, Emanueli C, Madeddu P (2010) Vitamin B1 analog benfotiamine prevents diabetes-induced diastolic dysfunction and heart failure through Akt/Pim-1-mediated survival pathway. Circ Heart Fail 3(2): 294-305. CIRCHEARTFAILURE.109.903450

[2] Jia G, Whaley-Connell A, Sowers JR (2018) Diabetic cardiomyopathy: a hyperglycaemia- and insulin-resistance-induced heart disease. Diabetologia 61(1): 21-28

[3] Katare R, Caporali A, Emanueli C, Madeddu P (2010) Benfotiamine improves functional recovery of the infarcted heart via activation of pro-survival G6PD/Akt signaling pathway and modulation of neurohormonal response. J Mol Cell Cardiol 49(4): 625-638. 10.1016/j.yjmcc.2010.05.014

[4] Franchi F, Knudsen BE, Oehler E, et al. (2013) Non-invasive assessment of cardiac function in a mouse model of renovascular hypertension. Hypertension research : official journal of the Japanese Society of Hypertension 36(9): 770-775. 10.1038/hr.2013.43

[5] Oltean S, Qiu Y, Ferguson JK, et al. (2015) Vascular Endothelial Growth Factor-A165b Is Protective and Restores Endothelial Glycocalyx in Diabetic Nephropathy. J Am Soc Nephrol 26(8): 1889-1904

[6] van den Berg BM, Vink H, Spaan JA (2003) The endothelial glycocalyx protects against myocardial edema. Circ Res 92(6): 592-594

[7] Singh A, Ramnath RD, Foster RR, et al. (2013) Reactive oxygen species modulate the barrier function of the human glomerular endothelial glycocalyx. PLoS One 8(2): e55852. 10.1371/journal.pone.0055852

[8] Singh A, Friden V, Dasgupta I, et al. (2011) High glucose causes dysfunction of the human glomerular endothelial glycocalyx. Am J Physiol Renal Physiol 300(1): F40-48. ajprenal.00103.2010

[9] Ramnath R, Foster RR, Qiu Y, et al. (2014) Matrix metalloproteinase 9-mediated shedding of syndecan 4 in response to tumor necrosis factor alpha: a contributor to endothelial cell glycocalyx dysfunction. FASEB J 28(11): 4686-4699. 10.1096/fj.14-252221

[10] Lin H, Suleiman MS (2003) Cariporide enhances lactate clearance upon reperfusion but does not alter lactate accumulation during global ischaemia. Pflugers Arch 447(1): 8-13. 10.1007/s00424-003-1134-8
